# Heuristics and semantic spaces for the analysis of students’ work in mathematical problem solving

**DOI:** 10.1007/s10649-023-10297-y

**Published:** 2024-02-15

**Authors:** Stéphane Favier, Jean-Luc Dorier

**Affiliations:** University of Geneva, Geneva, Switzerland

**Keywords:** Heuristics, Problem representation, Problem solving, Semantic space, Real class conditions

## Abstract

In this research, our objective is to characterize the problem-solving procedures of primary and lower secondary students when they solve problems in real class conditions. To do so, we rely first on the concept of heuristics. As this term is very polysemic, we exploit the definition proposed by Rott (2014) to develop a coding manual and thus analyze students’ procedures. Then, we interpret the results of these analyses in a qualitative way by mobilizing the concept of semantic space (Poitrenaud, 1998). This detailed analysis of students’ procedures is made possible by collecting audiovisual data as close as possible to the students’ work using an action camera mounted on the students’ heads. We thus succeed in highlighting three different investigation profiles that we have named explorer, butterfly, and prospector. Our first results tend to show a correlation with these profiles and the success in problem-solving, yet this would need more investigation.

## Introduction

Problem solving is at the heart of mathematical activity, and a great deal of research has been devoted to modeling the complex processes that someone mobilizes when solving a problem. These processes are studied through the main phases of solving and their sequence (Carlson & Bloom, 2005; Favier, 2022a; Mason et al., 1982; Pólya, 1957; Rott, 2012b; Schoenfeld, 1985) or through attributes that play an important role (Carlson & Bloom, 2005; Pólya, 1957; Schoenfeld, 1985) such as knowledge, heuristics, metacognitive activities or beliefs. Recent works such as those of Koichu et al., (2003, 2006) and Rott (2012a) bring the central role of heuristics back to the fore. In our research,^1^ we are interested in the role of heuristics in problem solving, but unlike almost all the above-mentioned research, which takes place in laboratory conditions, our study context is that of a real classroom during a problem-solving education session. Although the teaching of mathematical problem solving in the classroom has been the subject of a great deal of research over the past few decades (Kroll & Miller, 1993; Lesh & Zawojewski, 2007; Wilson et al., 1993), Lester and Cai (2016) point out that: “there remain far more questions than answers about this complex form of activity” (p. 118). Our research therefore contributes to this body of literature, and more specifically, our interest has been focused on problems that can be solved by the so-called trial-and-error method, which for us is characterized by the fact that students are led to make successive trial-and-error adjustments.

To present this empirical research, we will give some insights concerning the concept of heuristics and the concepts of representation of a problem and semantic space. Then, we will discuss the methodological elements related to the collection and processing of data before presenting the analyses and the results that we obtained.

## Theoretical background

### The concept of heuristics

Several studies use this concept in the problem-solving trend and propose different characterizations. Heuristics could be mental operations (Pólya, 1945), general rule (Schoenfeld, 1985), strategy or tactics (Larson, 1983), knowledge specific to problem solving (Julo, 1995) and even process (Posamentier & Krulik, 2009).

The differences also concern their properties. Heuristics could be linked with planning and control activities (Goldin, 1998). They can constitute the main structure or, on the contrary, a set of very local stages in the resolution process (Koichu et al., 2006). Lastly, the effectiveness of a heuristic is unguaranteed (Julo, 1995; Verschaffel, 1999). The point is clear, as Rott (2014) suggests: “despite its importance for problem solving (research), there is not a generally accepted definition of the term ‘heuristic’” (p. 176).

In addition to these conceptual elements on heuristics, we must now address the question of the operationalization of heuristics for the analysis of problem-solving processes.

In mathematics education, heuristics were first an object of study (Kantowski, 1974; Kilpatrick, 1967; Lucas, 1972) before becoming a tool, particularly in the context of an explicit teaching of heuristics. While such an explicit teaching has not produced the expected results (Schoenfeld, 1985), heuristics nonetheless remain a tool for analysing problem-solving processes (Carlson & Bloom, 2005; Koichu et al., 2006; Rott, 2012a, 2014, 2015). However, Rott (2015) points out that the wide variety of characterizations of heuristics leads to results that are difficult to compare. To overcome this pitfall, we have chosen to use Rott’s definition that makes a kind of compromise between all of these different characteristics put forward in his study:

Heuristics is a collective term for devices, methods, or (cognitive) tools, often based on experience. They are used under the assumption of being helpful when solving a problem (but do not guarantee a solution). There are general (e.g., “working backwards”) as well as domain-specific (e.g., “reduce fractions first”) heuristics. Heuristics being helpful regards all stages of working on a problem, the analysis of its initial state, its transformation, and its evaluation. Heuristics foster problem solving by reducing effort (e.g., by narrowing the search space), by generating new ideas (e.g., by changing the problem’s way of representation or by widening the search space), or by structuring (e.g., by ordering the search space or by providing strategies for working on or evaluating a problem). Though their nature is cognitive, the application and evaluation of heuristics is operated by metacognition (Rott, 2014, pp. 189–190).

Furthermore, in the fields of artificial intelligence and cognitive psychology, heuristics have been mobilized in connection with the concept of problem space to interpret certain phenomena observed in problem solving. We found it interesting to take up this concept in our own work, so we will now present this concept and explain how we propose to operationalize it in our analyses.

### Representation of a problem and semantic space

The notion of problem space has been introduced by Newell and Simon (1972) who proposed a new way of conceiving problem solving as moving within a problem space, which can be represented in the form of a graph. Each node in the graph represents a state that can be reached by the problem solver in the course of solving the problem. Richard (2004) defines this space as the result of the interpretation of the problem, which he calls the *problem representation*, and which comprises three components: the interpretation of the initial situation, the interpretation of the goal situation and the interpretation of the actions enabled by the problem. These three components depend on the problem, of course, but also on the problem solver. For a given problem, there is a problem space that corresponds to the real constraints of the problem, called the *task environment* by Newell and Simon, and the *effective space* by Richard. On another hand, the data in a problem may not be interpreted correctly. In such cases, the space in which the subject investigates is not the effective space, but what Poitreneau (1998) calls the *semantic space*. Here, it is interesting to realize that there may be a large number of semantic spaces, some of which may be smaller than the effective space, while others may be larger.

This distinction has been developed and formalized in the analysis of manipulation problems, of which the famous Hanoi Tower problem is emblematic. Nevertheless, Richard (2004) points out that this distinction between effective space and semantic space also applies to mathematical problems. In this sense, we see the link with the work of Julo (1995). Indeed, this psychologist is specifically interested in the construction of representations of mathematical problems. According to him, the construction of representations is mainly based on three processes:

- The process of interpretation and selection which involves what the wording of a problem communicates as a set of elements, the semantic context, the interpretation of which allows us to access “the information concerning the object and the task which characterizes the problem” (p. 31)*.^2^
- The process of structuring which is opposed to the idea of a progressive enrichment of the representation induced by the elements communicated by the problem wording. On the contrary, Julo invites us to consider that representations are “strongly organized entities” (p. 42)*. Their content is “a structured whole, i.e. the elements are interdependent and constitute a whole that has its own functioning and logic” (Ibid)*.
- The process of operationalization, which has the function of enabling “the transition to action, whether it be actual action (starting calculations, making a drawing, groping…) or mental action (making deductions, developing a plan, …).” (Ibid., p. 50)*. Thus, we hypothesize that the heuristics mobilized by the students that we can identify are visible traces of this process of operationalization of the representation. They make it possible to probe, at certain moments, the representation that the students construct for themselves during their solving.

According to Julo: “acting has many implications for the representation and is, presumably, one of the key drivers of its evolution.” (Ibid., p. 54)*. The evolution of the representation can result in the reinforcement of structuration or in “the implementation of a new structuration, but also the mobilization of new forms of knowledge” (Ibid., p. 55)*. This aspect is connected with the idea that the semantic space is dynamical and evolves according to what the problem solver is doing (Richard, 2004).

In summary, we consider that the semantic space characterizes a certain state of the representation that a problem solver constructs for himself of the problem he is solving. It contains the heuristics that embody the process of operationalizing this representation, as well as various other information that can be inferred about the processes of selection, interpretation, and structuring. Examples of how we operationalize this notion of semantic space are given in Sect. 3.

Finally, at the end of this literature review, we can formulate our research questions in the following way:

1. How can we use the notions of heuristics and problem space in order to analyze students’ procedures, working in groups, in real class conditions?
2. What role do heuristics play in the dynamics of the solving process?

In the following paragraph, we detail our research methodology.

## Methodology 

### Context and problem formulation

We conducted the experimental part of our research in six classes in three different grades of compulsory education in the canton of Geneva (2 classes per grade), in primary education: Grade 2 (age 7–8) and Grade 6 (age 11–12) and in lower secondary: Grade 8 (age 13–14).

We asked the teachers to manage the session in the usual way, and to leave students sufficient time to investigate the problem (about 45 min). They all chose to let the students work alone for a few minutes, then in groups of two or three.

On the other hand, we had chosen problems, one different for each grade (Table 1).

**Table 1:** Problems’ statement

Game of cards *(Grade 2)*	Each card in my deck represents either a triangle or a square. I pick 15 cards at random. I count all the sides of the figures drawn on the cards I picked and find 49. How many triangles and squares do you think I picked?
Dragons^a^ and co. (*Grade 6)*	On a computer screen are represented parrots, crocodiles and dragons. In total, I counted 20 heads, 72 legs and 30 wings. How many parrots, crocodiles and dragons are there?
Test ball (*Grade 8)*	In order to renew its sports equipment, a school makes a first order of 2 rugby balls, 4 basketballs and 4 soccer balls for a total amount of 72 CHF.^b^ It then places a second order for 2 rugby balls and 2 basketballs and pays 30 CHF. We know that a rugby ball, a soccer ball and a basketball cost 20 CHF together What is the price of each of the balls?

^a^A potential doubt about the number of legs of the dragons considered in this statement (four legs) was quickly cleared by the teachers

^b^CHF refers to Swiss francs

The students do not possess, even at grade 8, the algebraic tools for solving systems of linear equations. Therefore, in order to solve these problems, we expect them to engage in trial and error, and to take into account the result of one or more trials in order to propose another one and get closer to the solution until they find it.

### Data collection

In order to attempt to answer our research questions, we needed to collect experimental data as close to the students’ work as possible. To do this, we provided students with an on-board camera positioned on their forehead, which gave us access to their workspace from their own perspective. This is valuable in documenting the students’ problem-solving procedures.

Having students work in groups is a very common way of working in the classes and is a way to encourage oral exchanges. These verbalizations constitute valuable elements in the analysis of the students’ work.

In total, we recorded and analyzed the work of 17 groups of students in terms of heuristics: five for Grade 2, five for Grade 6 and seven for Grade 8.

### Data analysis

To process the audiovisual data collected in this way, we developed a coding manual. To do so, we started from the definition proposed by Rott (2014) presented above and, in support of our literature review, we listed the different heuristic proposals that could fit with this definition taken as a reference. The set of heuristics collected in this way constitutes the coding manual that we used to process our experimental data, an extract of which is given as follows (Table 2).

**Table 2:** An extract of the heuristics coding manual (The complete coding manual can be found in (Favier, 2022b))

Code	Description	Example
Changing the register of semiotic representation	Changing the register of semiotic representation from that (or those) of the statement or that already mobilized by the student in the first part of the resolution	Switching from a drawing to arithmetic writing
Making a trial	Proposing a possible solution to the problem	Considering and testing whether there can be 7 crocodiles, 5 parrots and 3 dragons
Working forward	Deducing certain information from the elements at disposal	Starting by deducing the number of wingless animals and then calculating their number of legs
Mathematical tool	Using a mathematical tool not indicated in the problem statement	Trying to use tools such as equations or proportionality
Symmetry property	Making use of the symmetry properties of the context or of the elements presented or encountered	For 15 animals with wings, consider the symmetrical cases: 13 dragons and 2 parrots or 2 dragons and 13 parrots

The analysis of our data also led us to enrich this list with some additional heuristics (Table 3).

**Table 3:** List of heuristics resulting from the analysis of our experimental data

Copying or highlighting certain data	Highlight certain data either by copying them or by highlighting them in the statement	Highlight data of the same nature in the same color
Introducing artifacts or material	Students take the initiative to use artifacts (compass, spreadsheet, …) or materials (tokens, multi-cubes, string, …)	Using one color of tokens for the squares and another for the triangles
Getting information (textbook, internet, …)	Students use other media to get information	Students consult their lesson books

With the support of this coding manual, we sought to identify, in our audiovisual data, the different heuristics mobilized by the students by characterizing them by their type and the moment of their manifestation. For the heuristics which are expressed over a long time, we chose it to be the starting point of the action or the verbalization which is selected. In addition, we distinguished between heuristics that were actually used by the students and those that were only mentioned but not used. It also happened that several heuristics were identified at the same time. Lastly, we identified any heuristics proposed by the teacher during feedback to each group.

Thus, we proceeded to a double-coding, i.e., the work of the different groups was coded by a research assistant and by us in an independent way. Then, we compared our coding results and proceeded to a joint coding to reach a consensus in the case of disagreements. The percent agreement (Jacobs et al., 2003, p. 100) for the identification of temporal cues (i.e., when a heuristic is identified) is 0.85 and 0.73 for the nature of the heuristics. This is quite acceptable and validates the methodology.

To carry out the analysis in terms of semantic spaces, we sought to infer, for each heuristic, complementary elements concerning the different processes involved in representing a problem. In particular, we sought to identify whether the problem solver incorporated an additional constraint to those communicated by the statement, or whether, on the contrary, he or she failed to take into account one or more of the indispensable data given in the wording of the problem.

Another important indicator concerns the registers of semiotic representation. Indeed, a change of register brings together different characteristics and potentialities in terms of representation structuring, which may lead the problem solver to investigate in different semantic spaces. Thus, a change of register could be equivalent to a change of semantic space.

Besides, using mathematical knowledge also has effects on the level of structuring and thus operationalizing the problem representation.

Finally, students’ beliefs about mathematics in general, problem-solving in particular, and the didactic contract (Brousseau et al., 2014) established by the teacher in the classroom can also influence their representation of the problem.

The various indicators we have just described are those we have used in our analyses, without claiming to be exhaustive. In retrospect, however, we realized that some of our choices were very close to the characteristics highlighted by Goldin (1998) in his systems of representation (external and internal). Moreover, we found a methodological similarity in the fact that describing the work of problem solvers using one or more representation systems always generates a certain ambiguity. Indeed, unlike analysis in terms of heuristics, which is based on more factual and therefore more objective elements, analysis in terms of semantic spaces seeks to infer what goes on in the problem solver’s head and is therefore much more subjective. Like Goldin, we therefore came to the conclusion that “with certain exceptions, *ambiguity* is a necessary feature in the concept of a representational system” (p. 145). The consequence of this state of things was the need for a single researcher to analyze and interpret all the data collected. In this way, we hope to reduce the risk of ambiguity through greater consistency and stability in the decisions made, and thus improve the perspective of the results obtained.

From a methodological perspective, we also have to mention that the analyses we carried out focused on the solving process by groups of two or more students, and not by single students, which was the case of most of the various studies mentioned above. At first sight, this might appear to be a limitation. However, our aim was to analyze work in real classroom conditions, and the group-work modality is very widespread (at least in the school contexts we frequented in France and in Switzerland). In this mode, the heuristics proposed by several students can be confronted, as can the elements of their representation. It seems to us that such analyses make sense, which is what we will illustrate in detail with two examples in the next section.

## Analysis

### First example: group 4PSJc12^3^

For each of the 17 groups, the first part of the analysis (the heuristics’ identification) is presented in the form of a table containing the time frame,^4^ the description of the action observed or the verbalization heard, and the title of the heuristic. For example, Table 4 corresponds to the work of a group of Grade 2 students on the *game of cards* problem.

**Table 4:**
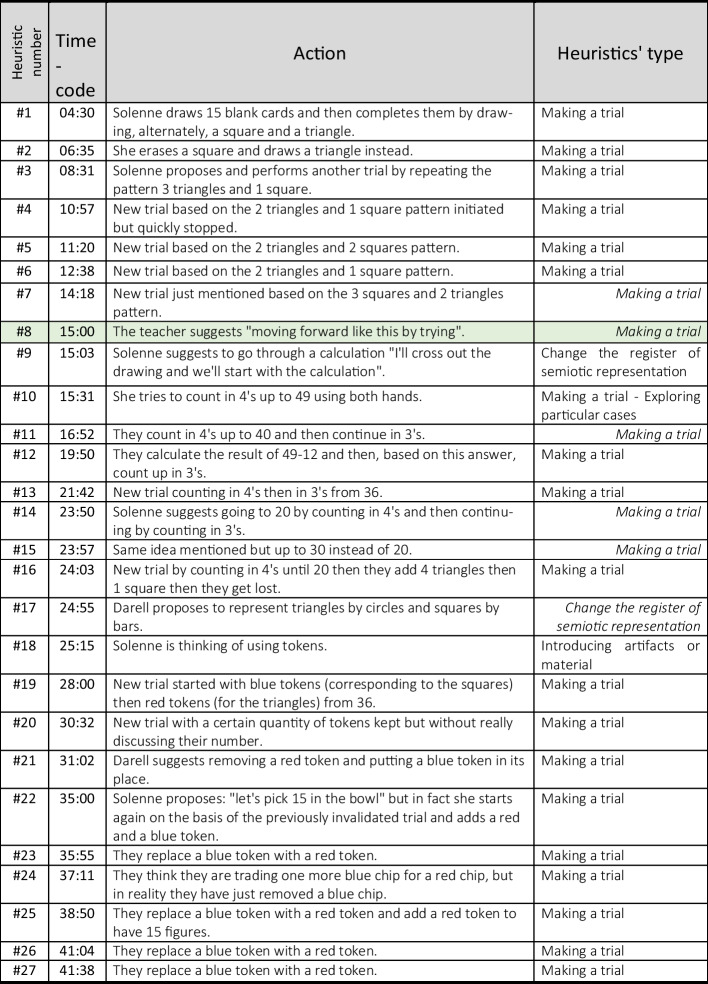
Heuristics analysis for group 4PSJc12

In order to distinguish the different cases of heuristics effectively implemented by the students or only evoked by some students or by the teacher, we played on layout effects. Thus, the lines on a white background represent the heuristics identified in the students’ work, while the lines on a green background correspond to those proposed by the teacher. The heuristics whose title is written in right-hand font aligned to the left in the cell are those that have actually been implemented. The heuristics whose title is written in italics and aligned to the right in the cell have only been mentioned, but not actually implemented. For example, at 12:38, the students perform a trial based on the repetition of the pattern “2 triangles and 1 square.” This trial was tested and invalidated, whereas the trial mentioned at 14:18 was only mentioned, but not actually tested afterwards.

It has happened, on several occasions, that different heuristics characterize what is done at the same time marker. This is, for example, the case at time 15:31, where not only does Solenne perform another trial (heuristic: making a trial) but additionally this trial is a special case, her counting in 4s indicating that she only considers four-sided figures (heuristic: exploring special cases).

**Fig. 1:**
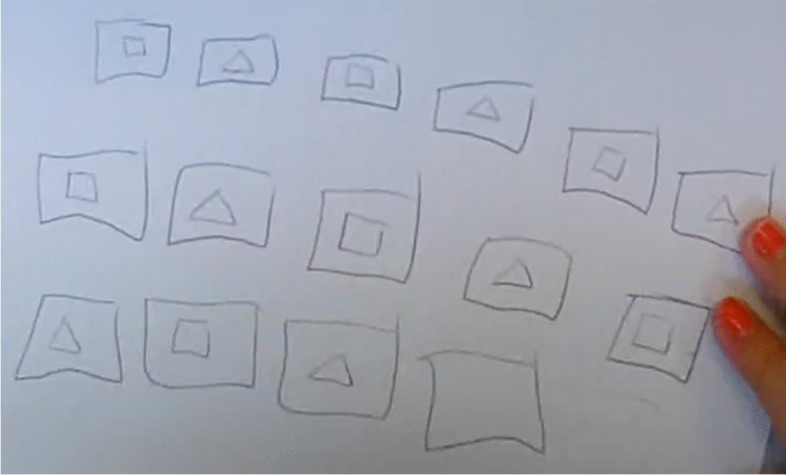
Solenne draws 15 blank cards and then completes them by drawing, alternately, a square and a triangle

**Fig. 2:**
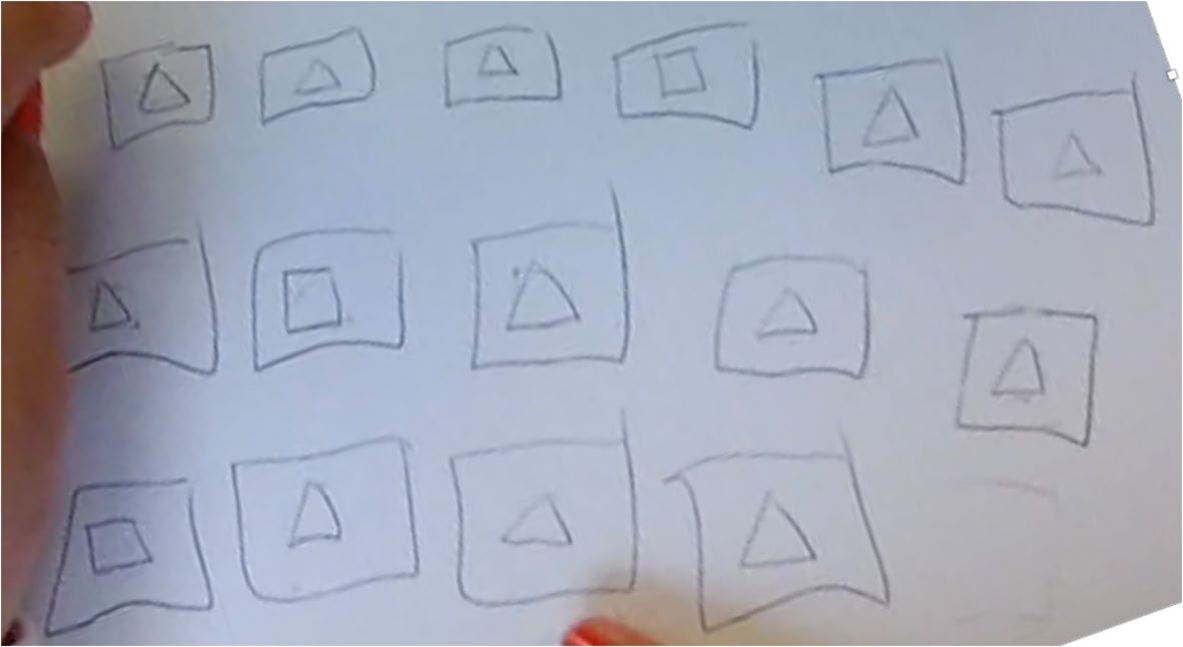
Solenne performs another trial by repeating the pattern three triangles and one square

For the second part of the analysis, we list the heuristics in their order of appearance and try to make visible for each some elements connected to the representation of the problem. For this duo in the first heuristic (#1), as can be seen in Fig. 1, Solenne draws 15 blank cards, which she then completes by drawing a square and a triangle alternatively, stopping when she has obtained 49 sides. We interpret the fact of drawing 15 blank cards as a way of taking into account the constraint that there are 15 cards. Stopping when getting to 49 sides shows that her representation of the problem takes the other constraint of the problem into account. On the other hand, another feature of the semantic space is the fact these students always alternate a square and a triangle, which is not a constraint of the problem but one that they impose on themselves with regard to the order in which the cards should be drawn. The implementation of the following heuristic (#2) is interrupted (at 6:35) by an intervention from the teacher, before the students can actually complete their idea. However, this does not seem to have any significant influence in the representation of the problem by the students. Indeed, it seems that, without any significant evidence of a contradiction, the new heuristic belongs to the same semantic space. Then, for the next heuristic (#3), Solenne proposes another trial, erasing the figures already drawn while keeping the cards. The photo in Fig. 2 shows that this trial is also based on the repetition of a pattern, this time made up of three triangles and one square.

In this way, we find the same characteristics (15 blank cards, alternating figures) of the semantic space in which Solenne carries out her investigation. The heuristics identified next (no. 4, no. 5, no. 6, and no. 7) display the same characteristics in terms of problem representation. In addition, we infer a further feature of this semantic space corresponding to the way in which adjustments are made. Indeed, adjustments from one trial to the next concern the composition of the pattern that generates the trial. These first seven heuristics are all ways of operationalizing the same representation of the problem. Therefore, this first semantic space is explored through these seven heuristics.

At 15:00, heuristic no. 8 proposed by the teacher does not encourage this representation to change, since the alternative constraint is not questioned and the teacher encourages the students to continue in this way.

**Fig. 3:**
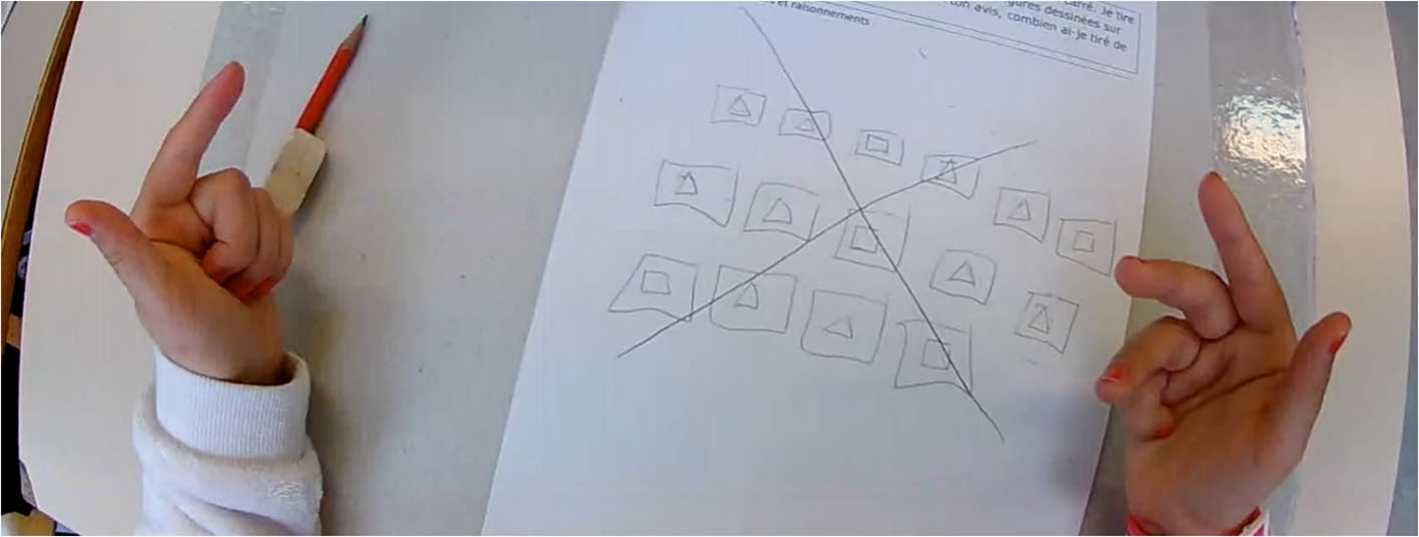
Solenne crosses out the drawing and goes through a calculation

Then, Solenne crosses out the last trial (Fig. 3) and suggests making calculations (no. 9). She uses one hand to count the number of figures and the other to count the number of sides as shown in Fig. 3.

In addition to the change of register, we note that Solenne counts in fours (i.e., squares), which reveals a significant difference from the first semantic space in the sense that the students abandon the constraint of the alternating figures. So, this evolution of representation and the exploitation of this new register characterize the opening of a new semantic space. The following heuristics (no. 10 to no. 15) correspond to trials carried out in the same way, i.e., students count in fours up to a certain number, and then continue by counting in threes. Note that adjustments are made when they move from counting squares to counting triangles. This aspect of the way of adjusting is clearly opposed to the first semantic space and is an additional feature of this second semantic space. We consider the following heuristic (no. 16) to have similar characteristics, even though the students fail to complete the trial. Indeed, the fact that they lose their way during the trial is undoubtedly a proof that they have reached the limits of this semantic space. However, this second semantic space is explored using these eight heuristics.

**Fig. 4:**
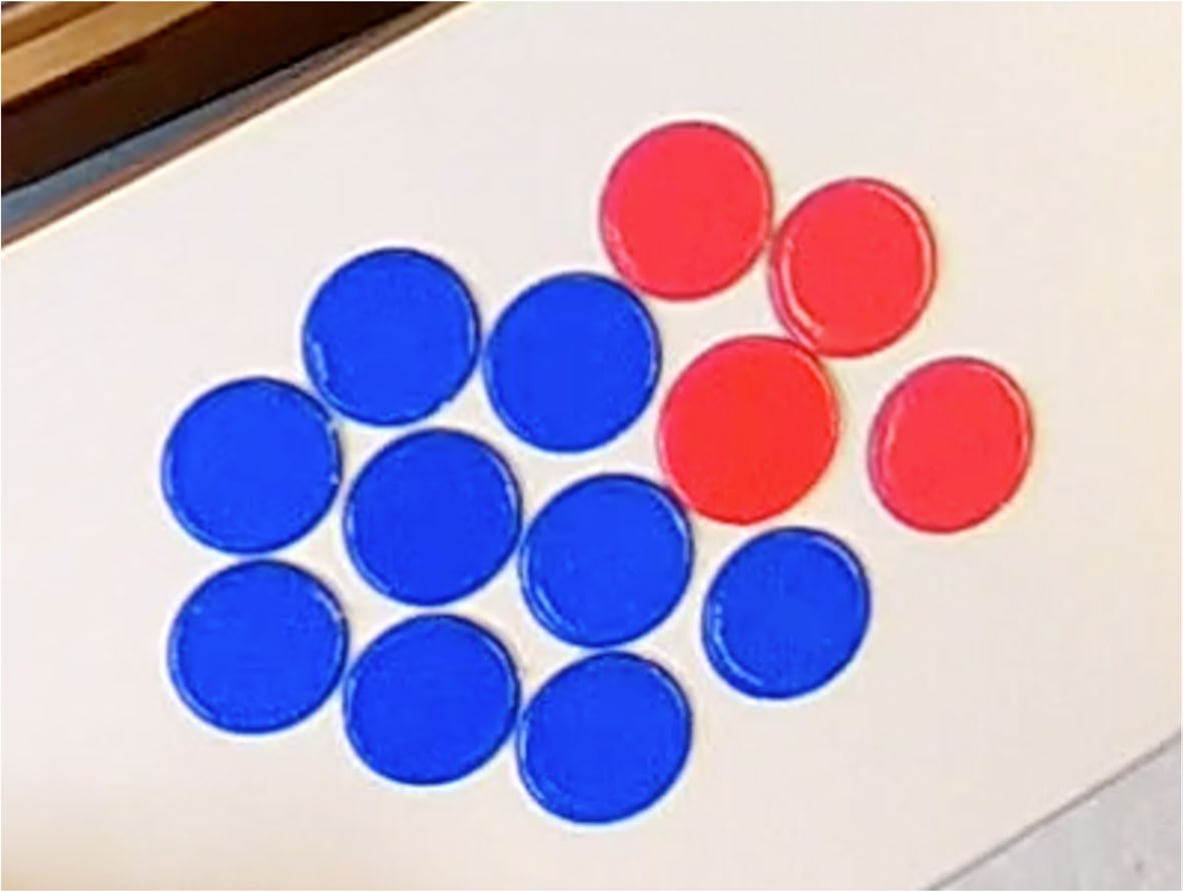
New trial realized with blue tokens (corresponding to the squares) and red tokens (for the triangles)

At 24:55, Darell suggests representing triangles with circles and squares with bars, thus introducing another change of register (no. 17). Although we have no further details, we can see here the opening up of another semantic space that will not be explored, since the students jump immediately to another idea and grab tokens (no. 18). They explain that squares are represented by blue tokens and triangles by red tokens. This new change of register also marks a new semantic space, the fourth for this group. Using this register, the students produce the trial represented on Fig. 4.

All the heuristics that followed until the end (no. 19 to no. 24) are linked to this representational register. We have to mention that during the implementation of some of these heuristics, the students made certain errors that can be linked to the nature of the manipulatives. For example, they picked up a token that had fallen on the floor and added it to the tokens making up their trial, even though it had come from their reserve. We can also see that for some trials, like the one shown in Fig. 4, the number of tokens in the trial does not sum up to 15. This shows a representation that does not simultaneously process both problem data, or at least does not take into account all of the problem constraints, as may have been the case in previous semantic spaces.

**Fig. 5:**
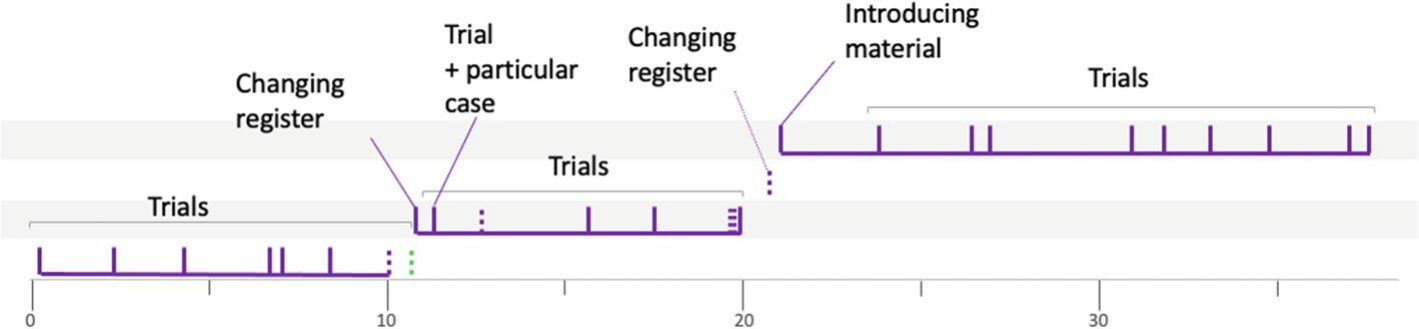
Solving procedure for group 4PSJc12

This progression through different semantic spaces is a way of analyzing the solving process implemented by the group of students. In order to have a more concise overview, we propose to represent it graphically as follows (Fig. 5).

The graduated axis represents time (in minutes). Each vertical segment symbolizes a heuristic, with a solid line if the heuristic has been implemented, or a dotted line if it has only been mentioned. A green dotted line represents a heuristic mentioned by the teacher. The horizontal lines represent the different semantic spaces. From the analysis described above, we can see that a first semantic space is explored during the first seven heuristics. Then, another semantic space is identified and explored during the next eight heuristics. Then, another space is identified, this one containing only one heuristic. Finally, the search continues in an even different semantic space, with the implementation of the last ten heuristics. The horizontal segment linking certain heuristics underlines the continuity of the search within the same semantic space.

The work of some other groups shows less continuity between the heuristics used like the work of the group 10LSBa12 solving the problem *Test ball.*

### Second example: group 10LSBa12

The list of heuristics implemented by this group is given in Table 5:

**Table 5:** Heuristics analysis for group 10LSBa12

Heuristic number	Time-code	Action	Heuristics’ type
#1	02:06	Janice introduces the notations *b* for basketball, *r* for rugby, and *s* for soccer	Introducing names and notations
#2	02:43	She writes the data of the problem in the form of equations	Making the link with a mathematical tool (theorem, property)
#3	05:25	Thomas explains his idea of combining (adding) the two orders. Same idea, written by Janice, but with a subtraction of these two orders	Working forward
#4	07:20	Zineb says that “it would be nice if they all cost the same” and then she proposes to calculate 20:3	*Working forward—Reducing the problem to an easier one*
#5	07:42	Janice proposes to work “by trial and error”	*Faire un essai*
#6	08:38	Janice begins to write the equation corresponding to the cumulation of the three data in the problem	Working forward
#7	09:55	After Janice mentions trial and error, Thomas suggests: “You do all the odd numbers below 10” and then Zineb writes the list of whole numbers from 1 to 14 and their double	Generate new data systematically
#8	11:41	Janice recalls the data of the problem and suggests: “we can already remove the 2 soccer balls on both sides”	Working forward
#9	15:15	From the list established by Zineb, they test the trial with *b* = 7 and *r* = 8, and *s* = 5 “we will do with the smallest we will do with 14 and 16”	Making a trial—Exploring particular cases
#10	17:16	Janice proposes the trial with *b* = 6 and *r* = 9 and *s* = 5	Making a trial
#11	17:28	Janice deduces that 1 soccer ball costs 5 chf	Working forward
#12	24:57	New trial proposed with *b* = 5 and *r* = 10	Making a trial
#13	25:31	Janice realizes that: “it can be either one or the other” meaning that either it is *b* = 5 and *r* = 10 or the opposite *b* = 10 and *r* = 5	Make use of the symmetry properties
#14	25:53	New trial with *r* = 4 and *b* = 11	Making a trial

**Fig. 6:**
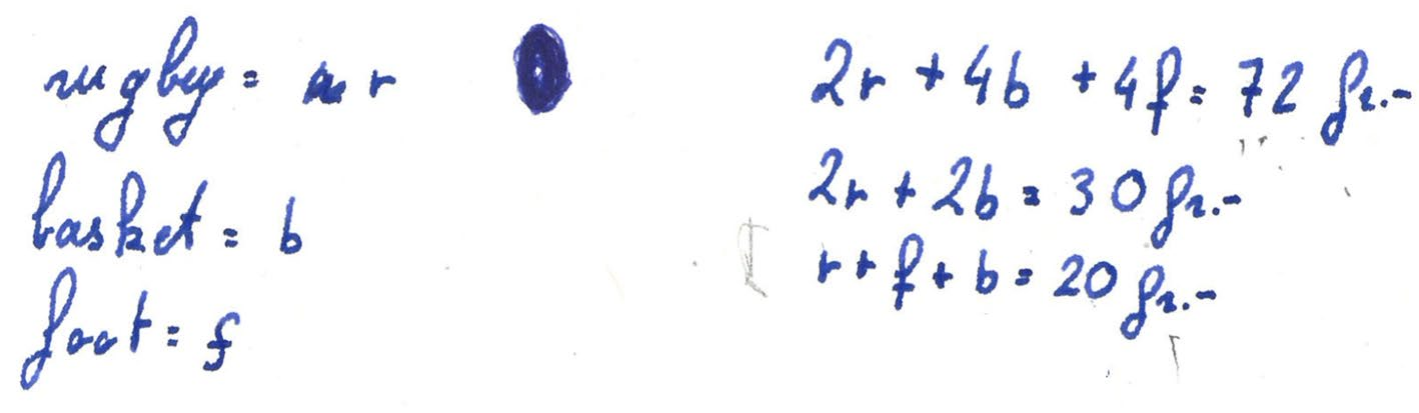
Janice translates the data given in the wording of the problem in an algebraic register

Janice begins her investigation by introducing a certain notation (no. 1) for the price of each of the three ball types. She then uses this notation to translate the data given in the wording of the problem in an algebraic register (no. 2, Fig. 6).

**Fig. 7:**
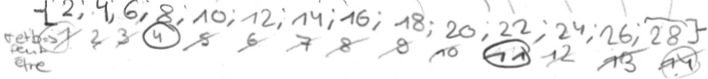
Zineb writes the list of whole numbers from 1 to 14 and their doubles

This first semantic space is characterized by the mobilization of the algebraic register and the taking into account of the essential data of the problem. After grouping, Thomas and Janice exchange their idea of combining some of the ball orders proposed in the wording of the problem (#3). We consider this to be another semantic space, as these students lack the knowledge required to solve a system of linear equations. Thus, the combinations of orders obtained seem to us to be the translation into algebraic language of manipulations carried out directly on the data of the problem (as opposed to a strictly algebraic treatment, which has been made without reference to the reality of the problem). The following heuristic (no. 4) is interpreted as taking into account an additional constraint, namely, the fact that the balls all cost the same. In our view, this strong constraint introduced by Zineb characterizes a new semantic space. This semantic space will not be explored anymore—indeed, the heuristic is only mentioned, since a few seconds later another heuristic (no. 5) reveals a break in the representation of the problem. Indeed, the idea of working through trials and adjustments reveals a different structuring of the representation, in that the data of the problem are not used to deduce new information, but to test whether the proposed values could be a solution to the problem. For us, this is a new semantic space that will not be explored immediately by the students, since after proposing this idea, Janice writes the equation corresponding to the accumulation of all the information given in the problem (no. 6). This heuristic shows similar characteristics to those of the second semantic space, to which we relate it. From the point of view of graphical representation (Fig. 9), this heuristic is therefore placed on the corresponding line, i.e., the second one. Again, this heuristic is not immediately followed by another belonging to the same semantic space, as the students list all the integers from 1 to 14 and their doubles (no. 7; Fig. 7).

There are several indications that this heuristic belongs to the fourth semantic space identified earlier (the same of no. 5). On the one hand, this list was produced after Janice’s reminder of the idea of working by trial and error. On the other hand, we can read on the left of this list “r et b peut être” which translates as “*r* and *b* could be” and shows that the students have listed all possible integer prices for rugby and basket balls. We can hypothesize that this list of possible values helps to structure this semantic space in a way that facilitates exploration. Graphically, this heuristic is placed on the fourth line from the bottom, which corresponds to the fourth semantic space identified during this resolution.

Then, Janice again suggests combining the balls orders (no. 8) that correspond to a heuristic with the same characteristics as those associated with the second semantic space.

**Fig. 8:**
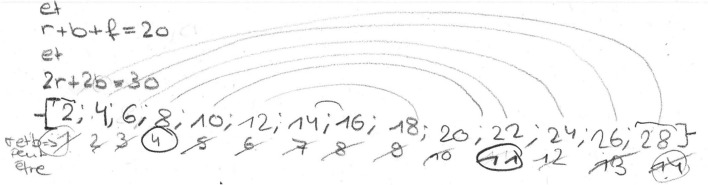
The connections between pairs of numbers

Then, using the list drawn up earlier, the students carry out two trials. These two heuristics (no. 9 and no. 10) are clearly linked to no. 5 and no. 7 and thus belong to the same semantic space, i.e., the same line on the graph. Following these trials, the students will notice a regularity in the price of the soccer ball and deduce that it is equal to 5 francs (no. 11) before proposing another trial (no. 12), namely, 5 francs for the basketball and 10 francs for the rugby ball. These two heuristics follow on from the previous ones and belong to the same semantic space. Finally, we interpret the following statement (no. 13) by Janice “it can be either one or the other” (meaning that it is either *b* = 5 and *r* = 10 or the opposite *b* = 10 and *r* = 5) as the exploitation of the register of semiotic representations mobilized, and in particular the connections between pairs of numbers made by Zineb (Fig. 8).

**Fig. 9:**
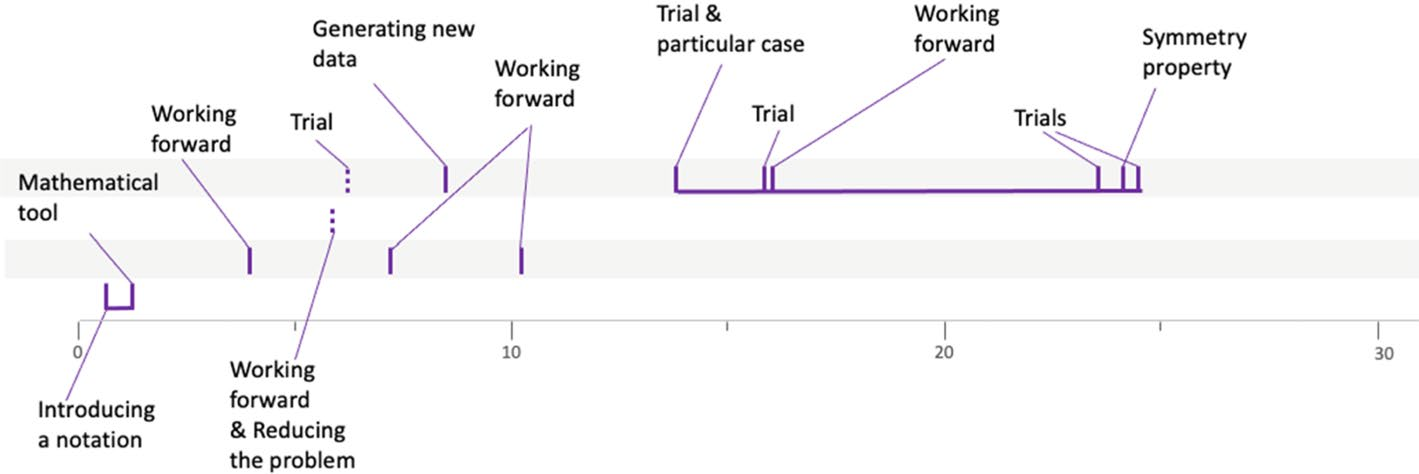
Solving procedure for group 10LSBa12

This gives birth to another heuristic (no. 14), which eventually leads to the solution of the problem. The representation of the problem will therefore not evolve any further, and we classify these last two heuristics in the same semantic space as the previous ones (Fig. 9).

To conclude with this example, we can see that the graphical representation clearly highlights the first part of the investigation, during which different ideas corresponding to different representations are confronted. We were able to identify four semantic spaces in this first part. In a second phase, the heuristics implemented all seem to be linked to the same representation of the problem, and therefore the same semantic space, which will undoubtedly play a decisive role in the discovery of the solution. After these detailed explanations of two examples, we now give in the following section an overview of the whole set of results obtained in our study.

## Results

The analysis of the students’ solving procedures in terms of heuristics and semantic spaces reveals significant differences according to two different criteria:

- the level of exploration of semantic spaces, according to the number of heuristics that are mobilized in the exploration
- the level of variability according to the number of semantic spaces opened

From the intersection of these two criteria, we can identify three profiles that we call explorer, butterfly, and prospector, which we will now characterize.

### The explorer profile

**Fig. 10:**
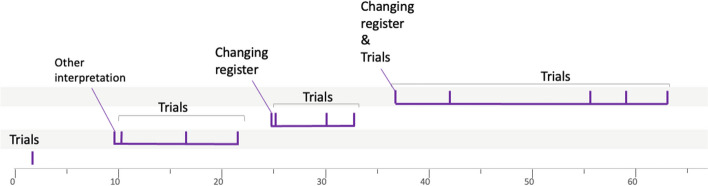
Solving procedure for group 4PCJc9

**Fig. 11:**
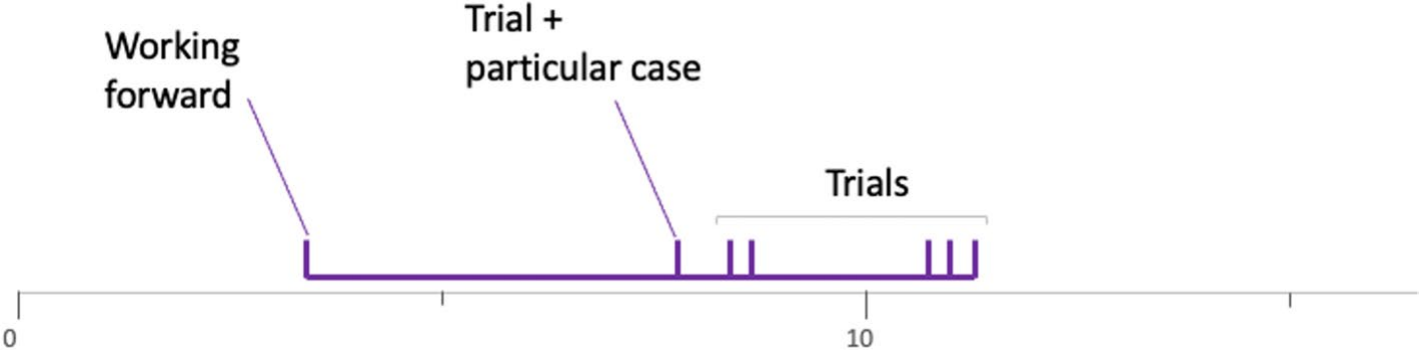
Solving procedure for group 8PSDr3

The explorer profile, of which the group 4PSJc12 (Fig. 5) is representative, is characterized by a relatively small number of open semantic spaces, of about 1 to 3, while each of these spaces is explored using several heuristics. For example, for group 4PSJc12, each semantic space is explored with more than six heuristics. Figures 10 and 11 show the procedure of two other groups characteristic of the explorer profile.

**Fig. 12:**
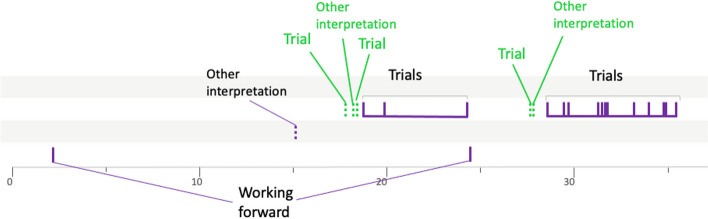
Solving procedure for group 10CTBa7

The explorers proved to be persistent since they used several heuristics before finding the answer (group 8PSDr3) or considering another semantic space (group 4PCJc9). For these three groups, as for groups 10LSBa14 and 8PVDr2 (Appendix 1), the investigation is not impacted by the teacher, i.e., the open semantic spaces are initiated by the students. This is not always the case since we can see in Fig. 12, which represents the procedure of group 10CTBa7, that the semantic space is opened by the teacher. In the rest of the resolution, it is this semantic space that is explored by the students. Then, not finding the solution to the problem, the students return to the first semantic space. It is again the teacher who brings the students back to the adequate semantic space previously considered. This time, it will be explored with the help of numerous heuristics.

**Fig. 13:**
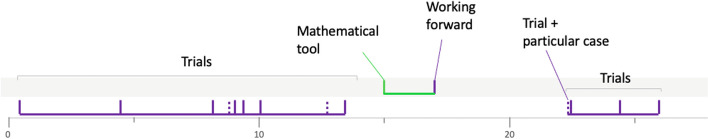
Solving procedure for group 4PCJc10

On the contrary, the explorers in group 4PCJc10 (Fig. 13) persist in exploring the first open semantic space. The teacher leads them to consider another semantic space which will be barely explored since the students return to their initial semantic space to continue their exploration successfully.

Appendix 1 presents three other groups in this explorer profile.

### The butterfly profile

**Fig. 14:**
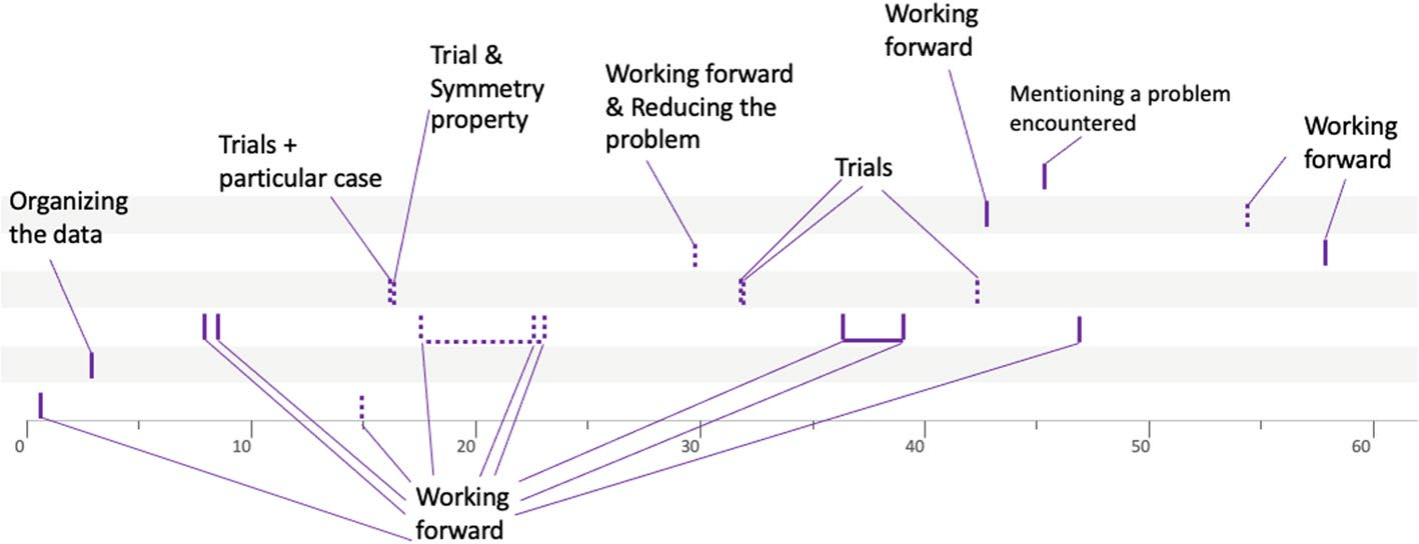
Solving procedure for group 8PVDr7

**Fig. 15:**
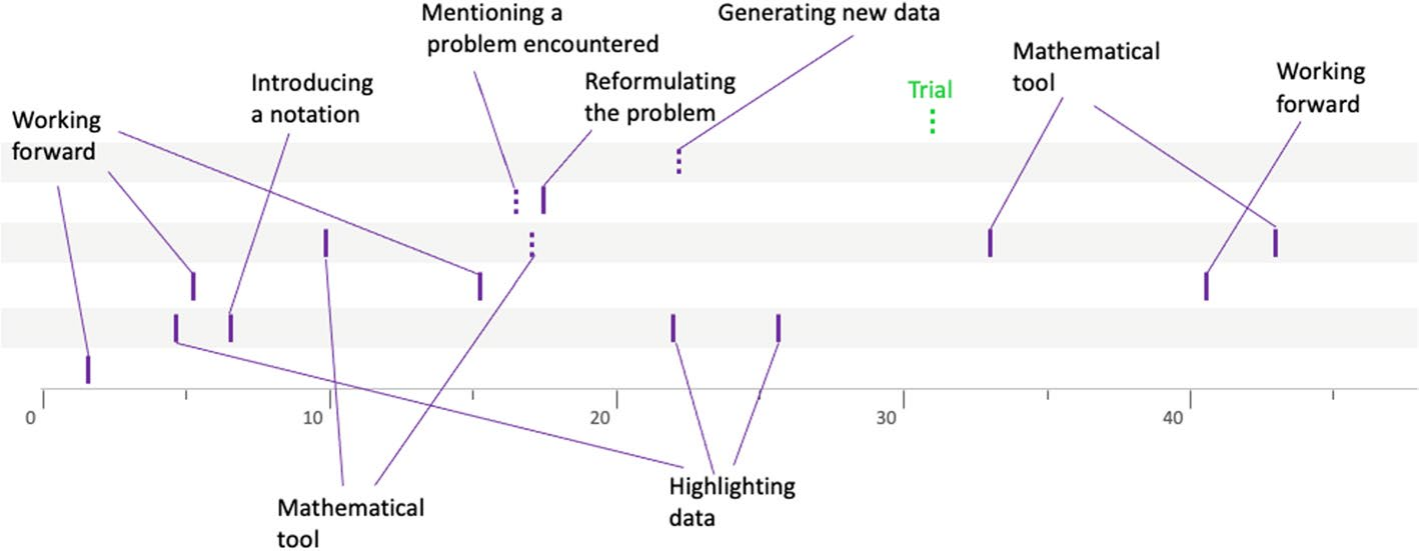
Solving procedure for group 10LSBa10

The procedures of groups 8PVDr7 (Fig. 14) and 10LSBa10 (Fig. 15) are representative of this profile. Indeed, several semantic spaces are open. This can be up to 7 in our data, but above all, what is very characteristic of this butterfly profile is that none of these semantic spaces are really explored. Even if several heuristics can appear in the same semantic space, there is no temporal continuity between them. It is as if the butterfly groups consider a semantic space and then open another one without having explored it. This description could seem negative, but it is worth noting that the butterfliy groups manage to open many semantic spaces, which points to a certain flexibility, a certain capacity to imagine different ideas.

Appendix 2 presents three others groups in this butterfly profile.

### The prospector profile

**Fig. 16:**
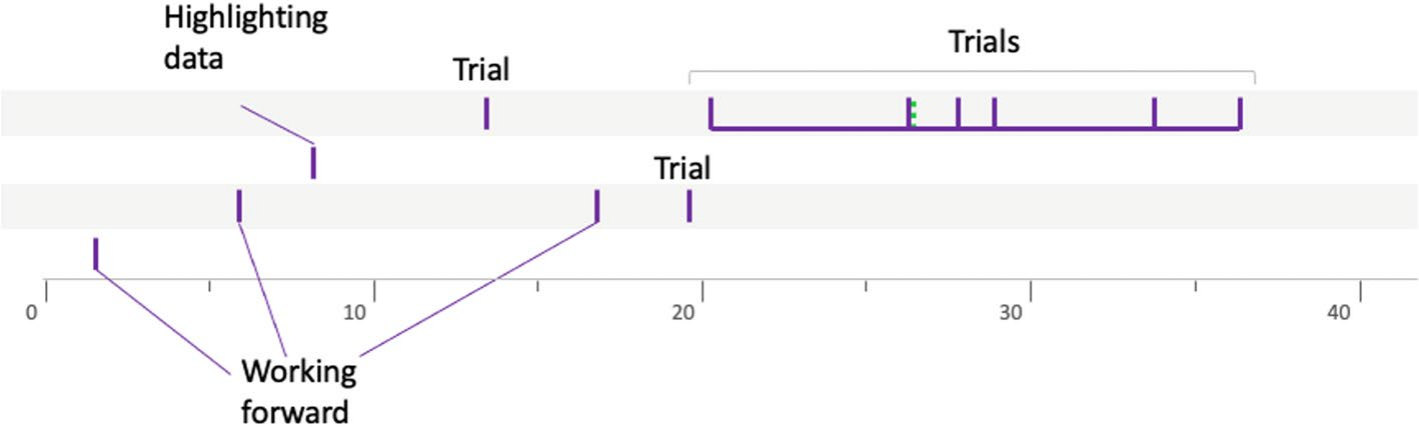
Solving procedure for group 10CTBa14

This profile appears to be a combination of the two previous profiles. Indeed, the prospectors go through a phase during which they consider different semantic spaces that they do not explore, before settling on a semantic space that they then explore using several heuristics. Group 10CTBa14 (Fig. 16) is representative of this type of procedure.

**Fig. 17:**
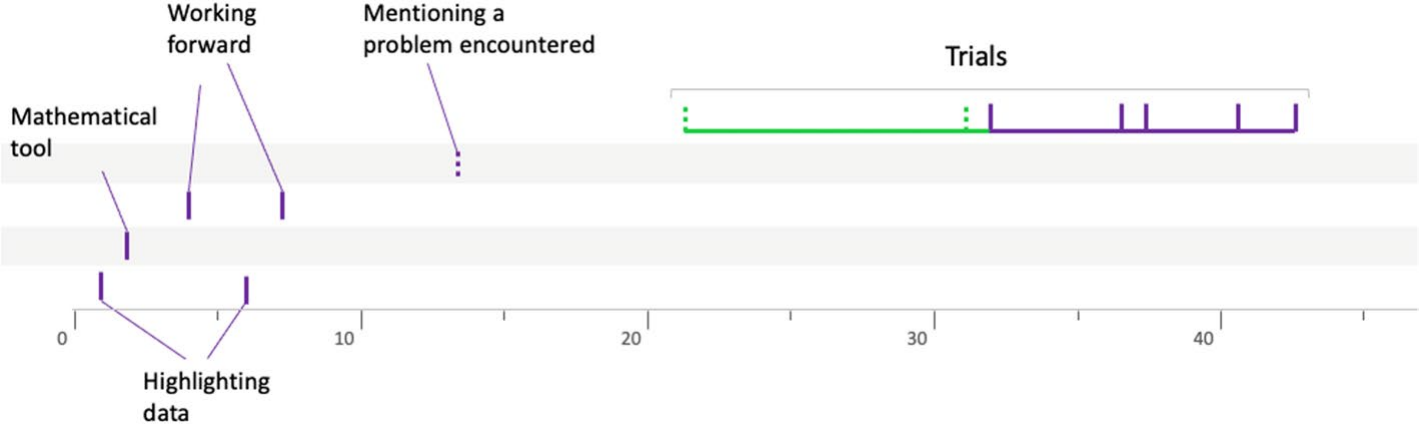
Solving procedure for group 10LSBa11

As for group 10LSBa11 (Fig. 17), it illustrates the case when the teacher opens the semantic space that will be explored by the students during their resolution. This is very different from the butterfly groups mentioned above whose students do not explore the semantic spaces opened by the teacher.

Appendix 3 presents one other group in this prospector profile.

### Link with the success in solving the problem

We sought to assess the possible link between success in solving the problem and these different profiles. To do this, we have evaluated and coded the success or non-success in solving the problem for each group of students. But as the work of some groups of students was modified or influenced by the teacher’s regulations, we ended up with four different levels to code the success in solving the problem:

- Right answer found without any help
- Right answer found with slight help
- Right answer found with strong help or guidance
- Right answer not found

To identify these four levels, we use the regulation analysis grid (Rott, 2020). The following table (Table 6) presents the distribution obtained.

**Table 6:** Distribution of the groups according to their profile and their success in solving the problem

	Right answer found without any help	Right answer found with slight help	Right answer found with strong help or guidance	Right answer not found
Explorer profile	4	2	1	1
Butterfly profile	0	0	2	3
Prospector profile	2	0	0	2

Thus, we see that four explorer groups find the answer without help, two groups with light help, one with strong help, and only one group does not find the right answer. For the butterfly profile, two groups find the answer with strong guidance, and the other three do not find the right answer. The prospector groups are evenly distributed since two groups find the answer without help, and two groups do not find the right answer. Thus, the groups that solve the problem are mostly in the explorer profile while those who do not find the answer are in the butterfly profile.

## Discussion

The link between problem-solving processes, heuristics, and problem spaces is not new. This has been an issue in psychology (Richard, 1994), where researchers used graphical representation of semantic spaces, which enabled them to visualize the subjects’ investigation and interpret certain phenomena in the light of certain heuristics. Our research had to face a new challenge due to the nature of the mathematical problems we have studied, which do not allow semantic spaces to be represented graphically, as for state-transformation problems studied by psychologists. Thus, the original contribution of our work lies in the fact that we have been able to establish a link between problem solving, heuristics, and semantic spaces entering through the analysis of heuristics and interpreting this analysis in terms of semantic spaces. To do so, we have focused on the processes involved in constructing the problem representation (Julo, 1995) and have operationalized several indicators: the identification of problem constraints added or omitted by the problem solver (Julo, 1995; Poitrenaud, 1998; Richard, 1994), the registers of semiotic representation (Duval, 1993; Goldin, 1998), the knowledge invested (Schoenfeld, 1985), and the students’ beliefs (Brousseau et al., 2014; Schoenfeld, 1985). This is what we have shown on the two examples detailed above, providing some answers to our first research question. Moreover, the use of resources and beliefs to interpret heuristics illustrates how the three components of Schoenfeld’s model (1985) can interact.

This analysis led us to identify different semantic spaces and to show that the problem-solving of these groups of students can be characterized by the number of semantic spaces they use, and by their level of exploration. We have identified three profiles, each featuring different specificities in terms of the problem-solving processes implemented. In this respect, there is a close connection with the heuristic behaviors proposed by Koichu et al. (2006), who also identified different student profiles based on different heuristic characteristics. However, our results are complementary to those of Koichu et al. because of the significant differences between the two studies, particularly regarding the methodology. For example, heuristics are not defined in the same way. Data collection using a thinking aloud method (in Koichu et al.) differs from our videos made with on-board cameras. Moreover, if Koichu et al. worked in clinical study conditions, each student being observed individually, we worked in normal classroom conditions, with students working in groups and interacting with a teacher. Nevertheless, it is interesting to see certain convergences in the different profiles issued of the two studies. We have seen that our explorer profile is characterized by few open semantic spaces. This profile seems to be similar to the progressive heuristic behavior profile of Koichu et al., where investigation is tightly focused on few attempts. Our butterfly profile has features in common with the circular heuristic behavior profile of Koichu et al. Indeed, these students make numerous attempts that become shorter and shorter as the solving progresses, which is likely to correspond to the opening up of numerous semantic spaces without any real exploration at the end. Lastly, students in our prospector profile are similar to those in the spiral heuristic behavior profile of Koichu et al. Indeed, both make numerous attempts, but above all, there is a progression in the number of attempts, like the prospectors, which ends up refocusing their investigation on a semantic space that they will explore in greater depth. Although these similarities must be put into perspective in view of the many differences that distinguish these two studies, it is interesting to note that these trends highlight different qualities. Perseverance dominates the exploratory groups, who explore semantic spaces in greater depth than the other profiles. The ability to imagine different avenues characterizes butterfly profile. Prospectors combine these two qualities. From the point of view of heuristics, and in an attempt to answer our second research question concerning the role of heuristics in the dynamics of problem-solving, analysis in terms of semantic spaces is a real added value. Indeed, it allows us to go beyond a mere quantitative analysis of the heuristics mobilized, as is generally the case in the research works on this topic. Indeed, on a more qualitative level, we can see here that the same heuristic can be used in one case to open up a new semantic space, and in another to explore it. The type of analysis we have set up gives in this sense a deeper perspective to students’ use of heuristics. Moreover, we confirmed results already found by several researchers (Kantowski, 1977; Koichu et al., 2007; Lucas, 1974; Rott, 2012a) showing that the number and diversity of heuristics mobilized is correlated with problem success. But we also showed through the success of the groups of the explorer profile that, to be more effective, the heuristics must also be used in greater depth and not just in a superficial manner like the butterfly profile.

However, the small number of participants in our study means that we need to be careful before drawing too generalized conclusions. It would be interesting to test these hypotheses on a larger scale and/or with other types of problem.

## Conclusion

First, from a methodological point of view, our research shows that our type of data collection and the quality of the data obtained are quite operational for identifying heuristics and interpreting them. It shows that it is possible to adapt elements of previous works to a real classroom context. In particular, it opens up new potentialities by overcoming some of the limitations of thinking aloud type data collection.

Moreover, the analysis in terms of heuristics is an interesting step to propose a qualitative analysis of the students’ problem-solving procedures, and the use of the notion of semantic space has proven to be efficient beyond the strict context of transformation problems.

Such an analysis led us to characterize different investigation profiles, which we have named explorer, butterfly, and prospector, based on the number of open semantic spaces and the level of exploration of each semantic space. When we cross the level of success of the groups in solving the problem with our three profiles, it is interesting to note that the explorer profile includes the majority of groups that succeed in solving the problem. Conversely, the butterfly profile has no groups that manage to solve the problem.

These results lead us to consider interesting research perspectives. Indeed, a further step would be to continue our research with a larger spectrum of problems and a larger number of students in order to verify if these profiles are still identifiable and if the success in solving the problem remains correlated with our three profiles. It would also be interesting to explore whether the repartition of profiles is more correlated with the groups’ grade or with the types of problem.

Furthermore, although various studies (Kantowski, 1977; Koichu et al., 2007; Lucas, 1974; Rott, 2012a) show that the use of heuristics is positively related to problem-solving performance, Schoenfeld (1985, 1992) puts them into perspective by specifying that the effects of teaching heuristics are relatively weak. Therefore, we do not plan to systematically teach heuristics to students in the classroom, but rather to consider heuristics as a tool for the teacher to regulate his or her interventions when students encounter difficulties. In the same way, being aware of the different types of semantic spaces, especially in relation to additional constraints that the students might impose on themselves, can be a relevant indicator in helping students solve problems and an element likely to complete the a priori analysis when preparing problem-solving sessions. These different aspects will be the focus of a new collaborative research project that we will conduct with primary and secondary school teachers.

## Figures and Tables

**Fig. 18:**
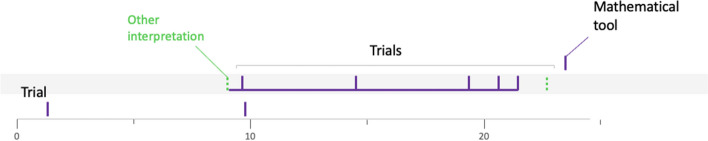
Solving procedure for group 4PCJc7

**Fig. 19:**
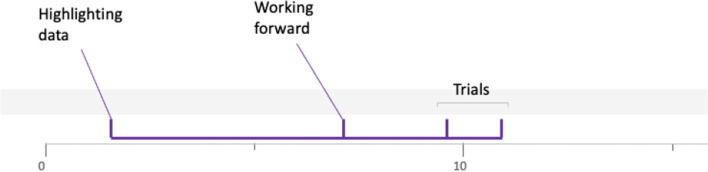
Solving procedure for group 8PVDr2

**Fig. 20:**
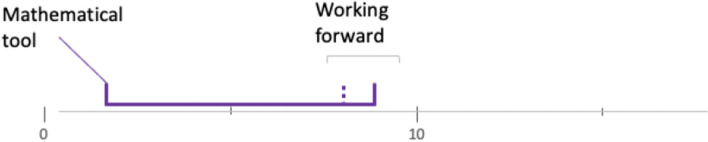
Solving procedure for group 10LSBa14

**Fig. 21:**
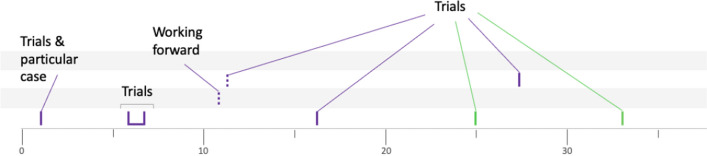
Solving procedure for group 4PSJc10

**Fig. 22:**
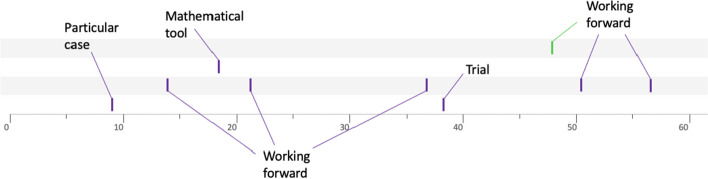
Solving procedure for group 8PSDr10

**Fig. 23:**
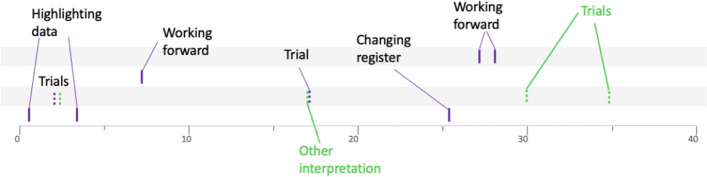
Solving procedure for group 10CTBa10

**Fig. 24:**
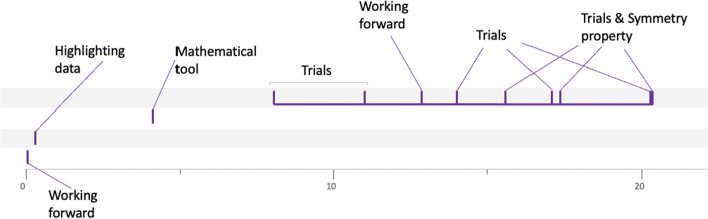
Solving procedure for group 8PVDr9

## Data Availability

The data that supports the findings of this study are available from the corresponding author upon reasonable request.

## Appendix 1. Other groups in the explorer profile

^18^, ^19^,^20^

## Appendix 2. Other groups in the butterfly profile

^21^, ^22^,^23^

## Appendix 3. Other group in the prospector profile

24

## Footnotes

1 This research is part of a more global project, financed by the Swiss National Science Foundation (SNF) entitled: *La résolution de problèmes comme objet ou moyen d’enseignement au cœur des apprentissages dans la classe de mathématiques**: **un point de vue fédérateur à partir d’études dans différents contextes*. Co-requestors Jean-Luc Dorier and Sylvie Coppé, Subside n°100019_173105 /1.

2 All quotations marked with an asterisk are our English translation of French original.

3 In this code, 4P indicates the class level in the Swiss system (grade 2); S designates the name of the teacher, Jc the problem at stake (“Jeu de cartes”, Game of cards), and 12 is the number of the on-board camera.

4 Note that the time corresponds to the time code of the whole video recording, i.e., it includes a certain duration between the start of the recording after the camera has been placed on the student’s head and the actual start of the student’s solving.
